# Liposarcoma of the spermatic cord: The state of art and our experience

**DOI:** 10.1016/j.radcr.2023.08.031

**Published:** 2023-08-30

**Authors:** Maria Antonietta Di Pilla, Marco Alex Capuano, Mariangela Rossi, Gianni Di Pilla, Rocco Minelli, Paolo Pizzicato, Antonio Rossi, Giuseppe Paviglianiti, Donatella Irace, Gianfranco Vallone, Antonio A.H. Salvia, Maria Cristina Smaldone, Valentina Cariello, Raffaele Zeccolini, Eugenio Rossi

**Affiliations:** a Life and Health Department “V. Tiberio”, University of Molise, Campobasso, Italy; b Department of Radiodiagnostics “F. Veneziale”, Molise Regional HealthCompany (ASREM), Isernia, Italy; c U.O.S.D. Diagnostic Imaging P.O. Pausilipon - AORN Santobono –Pausilipon, Naples, Italy; d Campus Biomedico University, Rome; e U.O.C. Pediatric Radiology PO G. Di Cristina-ARNAS Civico Benfratelli, Palermo, Italy.; f A.O.U. Federico II, Department of Translational Medical Sciences, Pediatric Section, Naples, Italy; g Department of Precision Medicine, University of Campania “Luigi Vanvitelli”, Naples, Italy

**Keywords:** Liposarcoma, Spermatic cord, Scrotal masses, Integrated imaging

## Abstract

Liposarcoma of the spermatic cord is a malignant neoformation so rare that less than 200 cases are reported in the world. It is a tumor that originates from adipose tissue and when it is found in the spermatic cord it can deceptively simulate an inguinal hernia and not be easily identified. The present work describes the case of a 37-year-old man with liposarcoma of the spermatic cord who arrives at our institution with painless swelling of the left testicle. Physical examination revealed a painless swelling in the scrotal sac. The scrotal ultrasound examination revealed a mass, measuring 8 cm (cranio-caudal) × 5.4 cm (latero-lateral) × 8 cm (antero-posterior) and characterized later with a basal CT examination of the abdomen. The patient was subsequently surgically treated with excision of the tumor, plus hernial plastic with plug and mesh. Histological examination revealed a mature adipocyte neoplasm whose morphological and molecular characteristics (amplification of the MDM2 gene) are consistent with the diagnosis of *dediferrentiated liposarcoma variety CO-MINGLED, G2 (sec. FNCLCC)*. The patient is currently under cancer surveillance with no signs of loco-regional recurrence. Spermatic cord liposarcoma is an extremely rare malignancy. It's not easy to identify as it can simulate an inguinal hernia, hydrocele, lipoma, funicular cyst, or testicular tumor. Diagnosis is usually established postsurgery, however, relapses are common and the role of chemo-radiotherapy remains to be defined.

## Background

The inguinal canal in males is a small canal in the lower abdominal wall, that contains the vas deferens, testicular artery and veins, lymphatic vessels, and nerves that make up the spermatic cord. The latter joins the epididymis and testicle within the scrotum and connects to the pelvic cavity. These structures can be involved in a wide range of diseases, including neoplastic, congenital, and inflammatory pathologies, which require a complete anamnesis and physical examination for diagnosis.

Spermatic cord liposarcoma is an extremely rare malignancy with fewer than 200 cases reported in world literature [1,2]. It is a tumor that originates from adipose tissue and when found in the spermatic cord, it can deceptively simulate an inguinal hernia and not be easily identified. These forms are more frequently found in the V-VII decade of life and often appear as asymptomatic, slow-growing, compact, and palpable paratesticular masses. The 5-year survival rate ranges from 15% to 85% depending on tumor grading, anatomic site, and ability to achieve full anatomic resection.

According to the WHO classification [1,3] of soft tissue cancers, liposarcomas are classified into 5 categories:

■ well differentiated, including adipocytic, inflammatory, and sclerosing subtypes;
■ de-differentiated;
■ myxoids;
■ round cell;
■ pleomorphs.

The histotypes associated with the worst prognosis are the round cell and pleomorphic ones, as they are associated with a higher rate of hematogenous recurrence and metastasis to the lung and bone. In contrast, differentiated and myxoid types are associated with loco-regional relapses [4,5].

Due to their rare occurrence, spermatic cord liposarcomas are often misdiagnosed as inguino-scrotal hernias, hydrocele, lipomas, funicular cysts, or testicular tumors [5,6]. Regarding the rarity of these tumors, there is little information on liposarcomas and there are no official recommendations and guidelines for the diagnosis, treatment, and follow-up of these patients.

Ultrasound is chosen as the primary imaging mode for its high sensitivity in characterizing intratesticular and extra-testicular lesions, but also for its ease of execution and availability, and its relatively low cost. Despite suggesting the diagnosis in only about 50% of cases, CT or MRI remain critical tools to guide the diagnosis and surgical planning for suspected malignant lesions within the groin region [5,6].

Definitive diagnosis through histological, immunohistochemical, and cytogenetic examination remains the gold standard. Classical histology consists of a sarcomatous, nonlipogenic, and usually high-grade cellular component.

## Case presentation

**Fig. 1:**
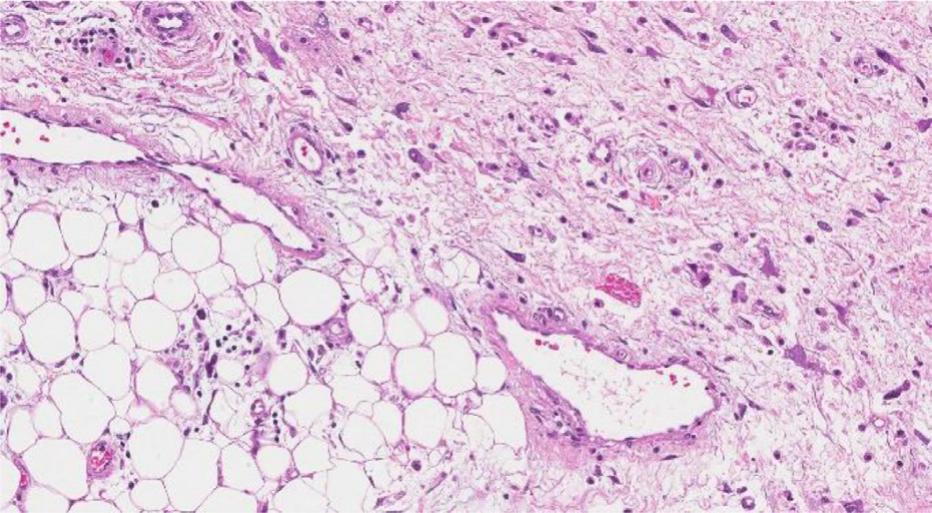
Dedifferentiated liposarcoma.

**Fig. 2:**
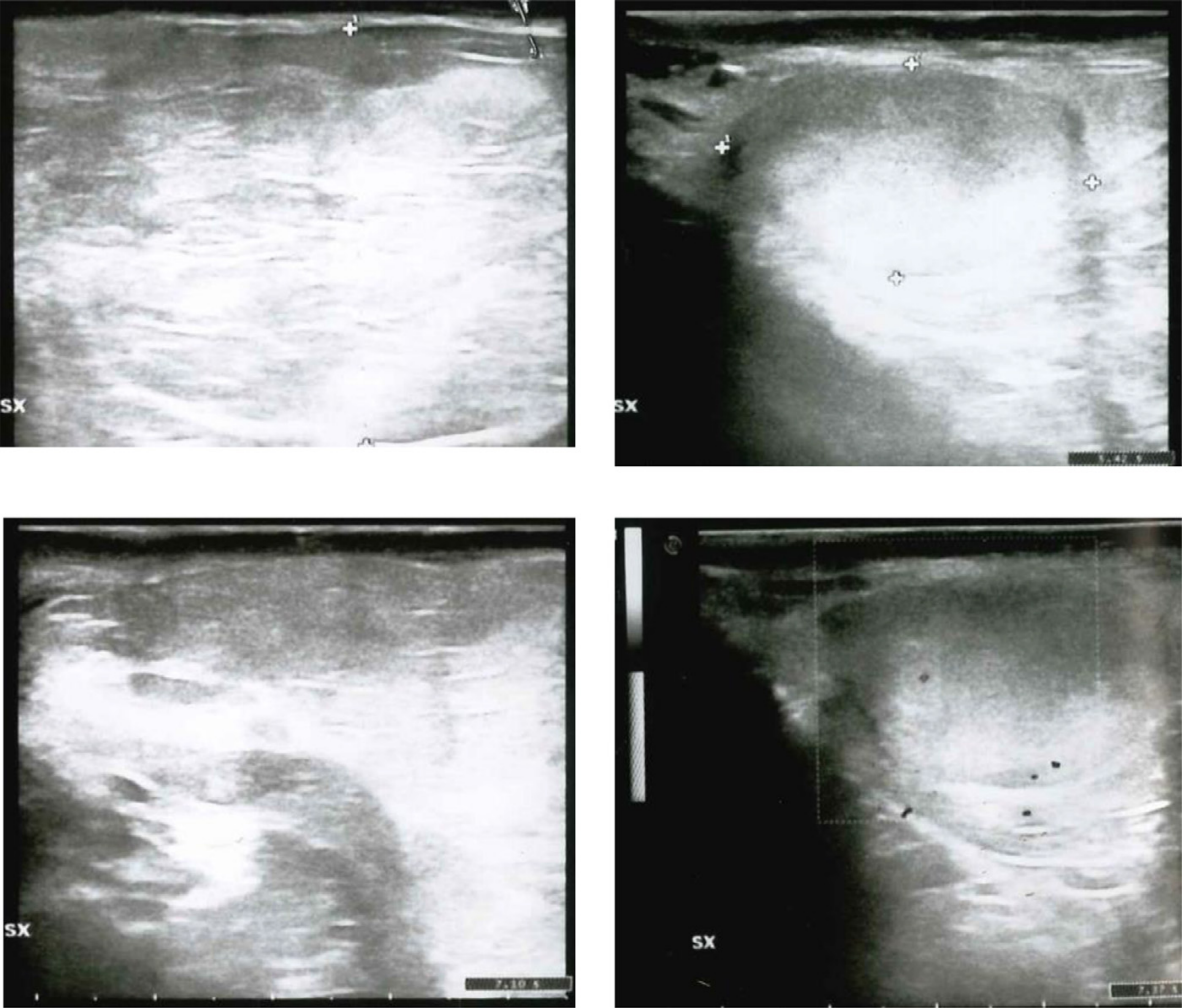
Ultrasound image of the lesion of the left spermatic cord (A and B); in homogeneously hyperechoic and poorly vascularized on Doppler (D). The above was accompanied by a left testicle of reduced size (C) compared to the controlateral (3.2 cm × 2 cm vs 4.8 cm × 2.4 cm) and with an in homogeneously hyperechoic echo structure as well as poorly vascularized (D) as from chronic tissue suffering.

**Fig. 3:**
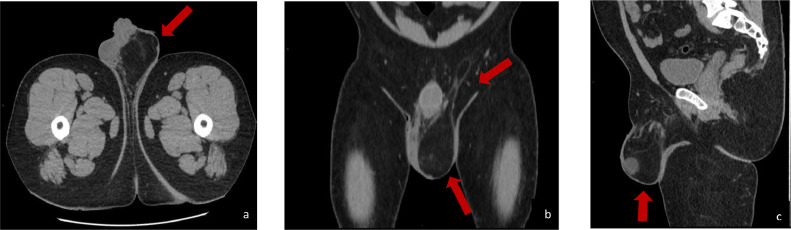
Transversal (A) coronal (B) and sagittal (C) CT scan of the mass of the left spermatic cord, which continued cranially with an adipose “cylinder” along the anatomical vector of the spermatic cord for a length of about 9 cm, of diameters 5.4 cm (lateral-lateral) × 8 cm (antero-posterior).

We describe the case of a 37-year-old man with liposarcoma of the spermatic cord who arrives at our institution with painless swelling on the left testicle. The family history highlighted a paternal grandmother who died of uterine cancer, a maternal uncle who died of gastric cancer, and a daughter in apparent good health. The patient is a nonsmoker and had a free and varied diet, as well as regular bowel and urination. Past medical history has underlined appendicectomy and hypofibrinogenemia not under drug treatment and an allergic diathesis to latex and iodinized contrast medium with vomiting and laryngeal edema. Physical examination revealed a painless swelling in the scrotal sac. The ultrasound examination (Fig. 1) showed a mass of the scrotal sac measuring 8 cm (cranio-caudal) × 5.4 cm (latero-lateral) × 8 cm (antero-posterior), disomogeneously hyperechoic and poorly vascularized on Doppler. The above was accompanied by a left testicle of reduced size compared to the counterlateral (3.2 cm × 2 cm versus 4.8 cm × 2.4 cm) and with a dismogeneously hyperechoic structure as well as poorly vascularized as from chronic tissue suffering. To better characterize the pathological findings yielded by the ultrasound, a basal CT examination of the abdomen was performed (Fig. 2). At CT scan, this pathological mass continued cranially with an adipose “cylinder” along the anatomical vector of the spermatic cord for a length of about 9 cm and with a maximum thickness of 1.7 cm, which gave reason for a plausibly “tilting” or “in the elevator,” in relation to the anamnestic relief of the onset of the swelling during the preceding 2 days. The above was accompanied by a left testicle of reduced size compared to the control one (3.2 cm × 2 cm vs 4.8 cm × 2.4 cm) and with an unevenly hyperechoic echo structure as well as poorly vascularized as from chronic tissue suffering. The right testis, on the other hand, appeared morpho-volumetrically within the limits and with a homogeneous echo structure, as well as normo-vascularized by color-Doppler. Elsewhere, the patient underwent surgical excision of the tumor plus hernial plastic with plug and mesh. Histologic examination resulted in a final pathologic diagnosis of *dediferrentiated liposarcoma variety CO-MINGLED, G2 (sec. FNCLCC)"* [2,7]*.* In particular, the examination showed a well-circumscribed nodular proliferation, with peripheral areas of increased cellularity, consisting of mainly mature adipocytes, mixed with elements with cytomorphological characteristics referable to lipoblasts. An abundant myxoid stroma and sometimes branched capillary structures were associated, while no areas of necrosis were found. The sample had the following immunohistochemical characteristics: S100 +, Cd34 -, MDM2 -, Actin ML -, Desmin -, Ki67 5% and mitotic index <1% per field. Currently, the patient is undergoing oncological follow-up by means of an MRI of the abdomen with and without paramagnetic contrast medium and basal CT of the chest, to exclude the appearance of metastasis (Fig. 3).

## Discussion and conclusion

In the adult population, over 75% of primary paratesticular cancers arise from the spermatic cord. Most authors agree that sarcomas originate primarily from mesodermal tissue rather than a malignant transformation of benign lipomatous tissue. Primary malignant tumors of the spermatic cord usually originate below the outer inguinal ring and therefore occur more often as scrotal masses than inguinal masses. The difficulties in diagnosing these tumors are due to the fact that they occur most of the time as painless scrotal masses; therefore, liposarcomas of the spermatic cord are often misdiagnosed as inguino-scrotal hernias, lipomas, hydrocele, epididymal cysts or testicular tumors. Most spermatic cord liposarcomas are well-differentiated low-grade malignancies with no or minimal tendency to metastasize. Liposarcomas are locally aggressive tumors with a high incidence of local recurrence. Given the numerous pathological conditions in the differential diagnosis of scrotal masses, the first level examination is usually ultrasound (US) [8]. The classic ultrasound presentation of spermatic cord liposarcoma is a heterogeneous and hypervascular mass with hyperechoic areas compatible with variable adipose/lipomatous levels within the tumor. Although ultrasound can provide useful information on the location, size, and consistency of the mass, there are no pathognomonic ultrasound features that can differentiate benign from malignant lesions, so second-level imaging is often required. Computed Tomography (CT) and MRI are superior diagnostic imaging tools useful for assessing the exact size, location, and tissue characterization of the tumor, as well as for assessing the condition of the spermatic cord and testis. According to some authors, these diagnostic methods may be useful in narrowing the differential diagnosis by suggesting fat-containing tumors such as liposarcomas. Definitive diagnosis is usually made after histopathological examination, considering that primary spermatic cord tumors do not show specific diagnostic imaging signs or patterns [4].

Radical orchiectomy, with high bone marrow ligation and large tumor resection, without positive microscopic margins is the essential element in the surgical management of primary spermatic cord neoplasms [8].

Given the high risk of postsurgical local recurrence, there is growing evidence that all spermatic cord tumors, regardless of their type and histological grade, are candidates for adjuvant radiotherapy, as they are often resistant to chemotherapy.

So spermatic cord liposarcoma is such a rare malignant growth that fewer than 200 cases are reported worldwide in the literature. It is a heteroplastic process that when found in the spermatic cord may not be easily identified by deceptively simulating an inguinal hernia, hydrocele, lipomas, funicular cysts, or testicular tumors. Diagnosis is usually established postsurgery, however, relapses are common and the role of chemoradiotherapy remains to be defined. The patients, therefore, undergo a careful follow-up for the prevention of recurrences, which are very frequent.

## Patient consent

With this item, we state that informed consent was acquired from our patient for inclusion in the associated study.
